# Model Input and Optimization: Improving the Speed and Accuracy of Our COVID-19 Hospitalization Forecasts

**DOI:** 10.1017/ash.2021.16

**Published:** 2021-07-29

**Authors:** Sarah Rhea, Emily Hadley, Kasey Jones, Alexander Preiss, Marie Stoner, Caroline Kery, Peter Baumgartner, Alex Giarrocco

## Abstract

**Background:** During the COVID-19 pandemic, public-health decision makers have increasingly relied on hospitalization forecasts that are routinely provided, accurate, and based on timely input data to inform pandemic planning. In North Carolina, we adapted an existing agent-based model (ABM) to produce 30-day hospitalization forecasts of COVID-19 and non–COVID-19 hospitalizations for use by public-health decision makers. We sought to continually improve model speed and accuracy during forecasting. **Methods:** The geospatially explicit ABM included movement of agents (ie, patients) among 104 short-term acute-care hospitals, 10 long-term acute-care hospitals, 421 licensed nursing homes, and the community in North Carolina. Agents were based on a synthetic population of North Carolina residents (ie, >10.4 million agents). We assigned SARS-CoV-2 infections to agents according to county-level susceptible, exposed, infectious, recovered (SEIR) models informed by reported COVID-19 cases by county. Agents’ COVID-19 severity and probability of hospitalization were determined using agent-specific characteristics (eg, age, comorbidities). During May 2020–December 2020, we produced weekly 30-day forecasts of intensive care unit (ICU) and non-ICU bed occupancy for COVID-19 agents and non–COVID-19 agents statewide and by region under a range of SARS-CoV-2 effective reproduction numbers. During the reporting period, we identified optimizations for faster results turnaround. We evaluated the incorporation of real-time hospital-level occupancy data at model initialization on forecast accuracy using mean absolute percent error (MAPE). **Results:** During May 2020–December 2020, we provided 31 weekly reports of 30-day hospitalization forecasts with a 1-day turnaround time. Reports included (1) raw and smoothed 7-day average values for 42 model output variables; (2) static visuals of ICU and non-ICU bed demand and capacity; and (3) an interactive Tableau workbook of hospital demand variables. Identifying code efficiencies reduced a single model runtime from ~100 seconds to 28 seconds. The use of cloud computing reduced simulation runtime from ~20 hours to 15 minutes. Across forecasts, the average MAPEs were 21.6% and 7.1% for ICU and non-ICU bed demand, respectively. By incorporating hospital-level occupancy data, we reduced the average MAPE to 6.5% for ICU bed demand and 3.9% for non-ICU bed demand, indicating improved accuracy. **Conclusions:** We adapted an ABM and continually improved it during COVID-19 forecasting by optimizing code and computing resources and including real-time hospital-level occupancy data. Planned SEIR model updates for enhanced forecasts include the addition of compartments for undocumented infections and recoveries as well as permission of reinfection from recovered compartments.

**Funding:** No

**Disclosures:** None

